# Towards a simple typology of international health partnerships

**DOI:** 10.1186/s12992-015-0132-x

**Published:** 2015-12-15

**Authors:** Suzanne Edwards, Dan Ritman, Emily Burn, Natascha Dekkers, Paula Baraitser

**Affiliations:** King’s Centre for Global Health, Weston Education Centre, Cutcombe Road, London, SE5 9JR UK; Tropical Health & Education Trust, 1 Wimpole Street, London, W1G 0AE UK

**Keywords:** International health partnerships, Typology, Training, Infrastructure strengthening

## Abstract

**Background:**

International health partnerships are one approach to capacity building in health systems. The evidence base for institutional partnerships for health service development remains weak and evaluation of the process and outcomes of health partnerships is a priority. The variability of partnerships contributes to the challenge of understanding their effectiveness and a typology of partnerships could aid evaluation. We analysed the proposals for all of the partnerships that received funding from the Tropical Health and Education Trust in 2012–2013 to develop such a typology.

**Methods:**

Our data consisted of 54 successful project proposals for health partnerships funded by THET in 2012–2013. A coding strategy was developed and modified through five rounds of coding, discussion, modification of the coding strategy and re-coding. The final coding strategy classified partnerships according to impact, approach and relationships between partners.

**Results:**

All 54 (100 %) of the partnerships in our sample planned to deliver training and 30 (56 %) aimed to deliver infrastructure strengthening in addition to training. 24 (44 %) aimed to build generic skills and 30 (56 %) specialist skills. 33(61 %) of the partners based in low and middle income countries had a scope of influence at national or international level and 33 (61 %) partnerships were between partners with an equal scope of influence. We suggest that those partnerships that focus on infrastructure strengthening and the development of generic skills might have more sustainable impacts in situations of high health care worker mobility and 12/54 partnerships met these criteria.

**Conclusion:**

We classified partnerships by their impact (scope of influence of LMIC partner and focus on individual/organisational development); approach to health systems strengthening (training/infrastructure; generic/specialist) and relationships (relative scope of influence between partners; mode of delivery – with an NGO partner or not). This is a first step in generating questions about partnership effectiveness that may be answered through evaluation.

**Electronic supplementary material:**

The online version of this article (doi:10.1186/s12992-015-0132-x) contains supplementary material, which is available to authorized users.

## Background

Health partnerships are one approach to capacity building in health systems. They aim to improve health services in low and middle income countries (LMICs) and the UK through long term, sustainable collaborations that involve the reciprocal exchange of skills, knowledge and experience between partners in the UK and overseas [1]. The evidence base for health partnerships as an approach to development remains weak and evaluation of the process and outcomes of health partnerships is a priority [2]. International health partnerships are highly variable and can relate to any aspect of capacity building in any type of health system [3, 4]. The variability of partnerships contributes to the challenge of understanding their effectiveness [2]. We anticipate that a typology of partnerships that provides a basis for comparing outcomes and approaches between groups of similar interventions would facilitate evaluation.

The Tropical Health & Education Trust (THET) has supported health partnerships for the last 25 years and is currently the managing agent for the Health Partnership Scheme (HPS), a United Kingdom Department of International Development- funded programme for health partnerships. The Health Partnership Scheme has awarded grants to 138 health partnership projects since its inception in 2011. In the first phase of HPS funding (grants awarded in 2012–2013 for projects of one to three years), THET awarded 86 grants across eight different grant streams: Multi-Country Partnerships; Long-Term Volunteering; Pilot Projects; Medium Paired Institutional Partnerships; Large Paired Institutional Partnerships; Volunteer Bursaries; Medical Equipment Partnerships; and Start-up grants. Grants ranged in size from £5000 to start a health partnership to £1.5million for a multi-country partnership.

We analysed the proposals of all of the partnerships that received funding from THET in 2012–2013 in 6 out of the 8 grant streams to develop a simple classification of health partnerships as an aid to describing and evaluating this approach. Excluded from the analysis were grants in the categories of Start-up (total of 26 grants) and Volunteer Bursaries (total of 4, all awarded to recipients of Large Paired Institutional Partnership grants) since the start up grants enable the setup of a partnership relationship only and the volunteer bursaries fund individuals to work within an existing partnership.

## Method

**Fig. 1:**
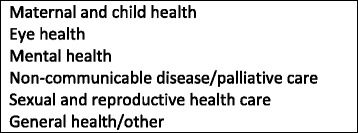
Early attempt to classify partnerships by clinical focus. Box containing text

Our data consisted of 54 of the 56 successful project proposals for health partnerships funded by THET in 2012–2013 out of a total of 378 submissions. Of the two that were not included, one did not accept the grant and the project did not go ahead, the other was excluded for administrative reasons. We used a framework approach to code the data [5]. This is an inductive process where the coding categories are generated directly from the data, in this case from the information within the partnership proposals. The initial coding categories were generated by reading and re-reading partnership proposals to identify an initial list of themes. We then reviewed the themes for their usefulness in grouping partnerships for the purposes of evaluation and comparison. The challenge was to code a set of highly variable partnerships in a manner that would both effectively describe this diversity and generate categories that could meaningfully group partnerships together. We therefore selected from our initial list of themes those that might impact on the partnership approach or outcomes. These decisions were made jointly by the research team with reference to published and unpublished evaluations of health partnership working and expert views. For example, an initial list of themes included ‘health care specialism’ and an initial coding category was generated for this theme (see Fig. 1). However this theme failed to support any further analysis or understanding of how or why partnerships might be similar or different in terms of their approach or outcomes and this category was removed from the framework. Expert views were obtained by consulting the THET Community of Practice for Health Links, an online discussion forum for those interested in or involved with partnership delivery, for ideas on useful categories for a typology of partnerships.

Once the initial themes were identified through the process of reading and re-reading and the sensitisation of the initial list of themes we generated a coding strategy for each theme that was developed and modified through five rounds of coding, discussion, modification of the coding strategy and re-coding. At key stages of the coding process 20 % of the coded data was checked for consistency by double coding, which was carried out blind by pairs of researchers (SE, ND, EB) with differences resolved through discussion. Detailed inclusion and exclusion criteria for each category were developed. For example the ‘education and training’ category included; ‘side by side training’, mentoring, buddying, clinical specialist training, visits by LMIC partners to UK for education and training and training of trainers but excluded the development of new curriculae. Similarly, the category ‘infrastructure’ included curriculum development, equipment supply, development of protocols and referral pathways and the development of new services. Our final coding framework is described in Table 1.

**Table 1:** Final coding framework

Theme	Category	Sub-categories	Description
Impact	Highest level of focus	Individual/organisational	If the intervention was provided so that the impact remained within the organisation after individuals had moved on, then projects were classified as having organisational level impacts. If the impact would move with health professionals as they moved between jobs then the impact was classified as individual.
	Absolute scope of influence of LMIC partner	Local/regional/national/international	Absolute scope of influence provided an estimation of size of the impact of interventions.
Approach	Strategy for capacity building	Education and training/infrastructure development	Approach to capacity building predominantly education/training or infrastructure strengthening.
	Health care issue addressed	Specialist/generic	Area of health care was classified as specialist if skills were not transferable to another area of clinical practice (e.g., fistula repair), and generic where they were (e.g., teaching skills for educators).
Relationship between partners	Mode of delivery	Direct delivery/via another organisation	Work was either delivered directly by one or other of the partner organisations or indirectly via a third organisation, a non-governmental organisation or a charity.
	Relative scope of influence of partners	Equal/unequal	Partnerships were classified by comparing the absolute scope of influence of the two partners.

## Results

### Highest level of focus

Half of the partnerships planned to have their highest level of focus at an individual level and half at an organisational level as shown in Table 2.

**Table 2:** Highest level of impact

Highest level of impact	Number (%)	Examples
Individual	27 (50.0 %)	Training provided by UK obstetricians, anaesthetists and paediatricians to doctors, nurses, midwives, nurse midwife surgeons and nurse anaesthetists in emergency obstetric care and anaesthesia including training of trainers
Organisational	27 (50.0 %)	Development of a new module in the undergraduate curriculum for the management of burns cases

### Capacity building strategy

All 54 (100 %) of the partnerships in our sample planned to deliver education and training and 30 (56 %) aimed to deliver infrastructure strengthening in addition to training.

### Health care issue addressed

Twenty-four partnerships focused on generic skills or activities, transferable across a number of health care tasks such as effective hand washing to improve infection control procedures, while 30 focused on specialist skills such as a specific surgical procedure that may be applicable in one context only (Table 3).

**Table 3:** Training focused on the development of generic or specialist skills

Approach	Number (percent)	Examples
Generic	24 (44.4 %)	A partnership between a health education institution in the UK and health education and delivery institution in LMIC with the aim of reducing neonatal mortality and paediatric infection through improved patient safety . UK partner provided training in the concept of patient safety; infection prevention, surveillance, management and treatment of common infections; how to collect data and use it to monitor trends in infections and how to use it to improve patient outcomes.
Specialist	30 (55.6 %)	A partnership between an NHS trust hospital in the UK and a health training institution and hospital in LMIC with the aim of strengthening capacity to provide training services for obstetric fistula repair and care of women living with fistulae. UK partner provided training of surgical teams and train the trainer workshops.

One of the aims of the health partnership scheme is sustainable capacity strengthening and we hypothesized that in situations of high healthcare worker mobility sustainable programme impacts would be associated with the development of capacity that would remain within the organisation after individual health care workers had left and generic health care worker skills that would be transferable between roles. On this basis we classified partnerships in four groups:

- Those focused on individual capacity building and generic skills – moderate sustainability
- Those focused on individual capacity building and specialist skills – lower sustainability
- Those focused on organisational capacity and generic skills – higher sustainability
- Those focused on organisational capacity and specialist skills – moderate sustainability

Table 4 gives the number of partnerships in each of these categories and examples of the work they were planning to deliver.

**Table 4:** Classification of partnerships to predict sustainability in situations of high healthcare worker mobility

12 partnerships focused on individual capacity building and generic skills	15 partnerships focused on individual capacity building and specialist skills
For example, a partnership between professional associations. Main activities were delivery of training courses in management of surgical emergencies, basic surgical skills, theatre nurse training and training the trainers. Intended outputs were 230 healthcare workers trained in emergency surgery, 120–200 theatre nurses trained in theatre and recovery techniques, approximately 50 surgeons trained to become trainers. Equipment for the courses left in place for future training (an element of infrastructure, but not the main focus of the intervention).	For example, a partnership between a health education institution and a health education and delivery institution in a national hospital. Main activities were delivery and evaluation of hands-on endoscopic therapy training course; six-monthly mentoring visits to consolidate training; training gastroscopy trainers programme. Intended outputs included courses delivered on management of acute upper gastrointestinal bleed and training of trainers.
12 partnerships focused on organisational capacity building and generic skills	15 partnerships focused on organisational capacity building and specialist skills.
A partnership between a professional association and a university hospital delivered courses in surgical safety; provision of equipment and educational materials; involvement of LMIC in quality improvement initiatives to develop leadership and clinical governance skills. Intended outputs were consistent use of WHO checklist; improved team-working and communication in operating theatres; improved strategies for patient assessment and management of perioperative critical illness; improved leadership and crisis response management in the theatre environment.	Partnership between a government teaching hospital in LMIC and a group of 15–20 health professionals from a strategic health authority area in the UK with the aim of improving multidisciplinary managements of stroke patients. Main activities were development, embedding and dissemination of core clinical skills identified as crucial for care of stroke patients in: swallow and nutrition, positioning and handling, communication and continence management. Methods used were educational sessions, mentoring and observational visits to the UK. Intended outputs were trained health professionals in the core stroke skill areas, families of patients attending group family education session and manual handling equipment in place.

### Absolute and relative scope of influence predicted impact of each partner

The absolute scope of influence of the activities of each partner provided an estimation of the size of the predicted impact of the partnership interventions. Our definition considered the scope of influence prior to the partnership. For example, a local hospital has a specific sphere of influence as an actor in a local health economy and the results of health system strengthening are likely to impact within this health economy in a way that is distinct from the international connections that come from partnership involvement. Similarly a university that delivers internationally recognised degree programmes and publishes research in peer reviewed journals has an international scope of influence that is distinct from its involvement in an international health partnership.

We classified absolute scope of partners as national/international/regional/local as follows: ‘International’ included research institutions with international activity; central governments; international professional associations such as the College of Surgeons of East, Central and Southern Africa. ‘National’ included national referral hospitals; ministries of health; national laboratories and national colleges. ‘Regional’ included district hospitals and district health boards. ‘Local’ included local hospitals, clinics and health boards; community groups and local training colleges. Thirty-three partnerships (61 %) had a scope of influence at national or international level, shown in Table 5. We hypothesised that international institutions would have a better sense of the importance of infrastructure, hence would be more likely to use a training and infrastructure approach than a purely training one. A chi-squared test for association showed that there was no statistical evidence of an association between absolute scope of influence and strategy for capacity building, X^2^ = 2.0, *P* > 0.5. Data are available in Additional file 1.

**Table 5:** Absolute scope of influence of low or middle income country partner

Scope of influence	Number (percent)	Examples
International	14 (25.9 %)	Partnership between NICE International and central government in China, Ministry of Health, Ministry of Social Security, National Health Resource Centre in India.
National	19 (35.2 %)	National referral hospital and National Public Health Laboratory
Regional	17 (31.5 %)	District referral hospital
Local	4 (7.4 %)	Health care complex consisting of a 200 bed general hospital with some specialist services

Relative scope allowed us to predict the relative influence of partners in relation to each other, a factor that may influence mutual accountability and collaboration. The majority of partnerships (61 %) were based on equal scope as determined by the level of the institutions involved, such as tertiary referral hospital to tertiary referral hospital, or professional association to professional association, and the job titles of the project leaders. The low income partner was more influential in 11 of the 21 unequal partnerships. Examples are given in Table 6. We hypothesised that relative scope of influence was more likely to be equal for partnerships where the LMIC partner had international/ national absolute scope of influence compared to regional/ local. There was no statistical evidence to support this, X^2^ = 0.8, *P* = >0.3 from chi-squared test. Data are available in Additional file 2.

**Table 6:** Relative scope of influence of partners

		Examples
Equal	33 (61.1 %)	Tertiary referral hospital in UK and in LMIC
		Professional associations of surgeons in both countries
Unequal/ Not clear	21 (38.9 %)	University hospitals & hospices in UK; Cancer Centre Welfare home and Research Institute in India [health delivery institutions]. UK partners have larger scope of influence, multiple hospitals, some with an international reputation compared to LMIC partner that is a single organisation with a 14 member team providing palliative care.
		University hospital department in UK and regional professional body in LMIC. Scope of influence is unclear, the professional body is an international organisation that covers a region with a population of 275 million. UK partner is a university with a world class reputation.

### Mode of delivery

The majority of partnerships, 69 %, managed and delivered interventions directly compared to 22 % who operated with a non-governmental organisation or a charity. Fourteen (26 %) operated across more than one LMIC.

## Discussion

We developed a typology of health partnerships and were able to classify all 54 partnerships funded by THET in 2012 using this typology. We completed an inductive analysis starting from descriptions of the partnerships found in their funding applications. The main challenge in this process was to go from the variability in the size, location, clinical specialty and approach of the individual partnerships to a simple classification that identified meaningful differences between partnerships as an aid to comparison and evaluation. Particular challenges included distinguishing between international and national partners as many national organisations will have some activity at an international level, for example ministries of health. To overcome this we categorised based on their day to day activities.

Previous attempts to classify health partnerships have described their area of activity. For example, a recent evaluation of the THET health partnership scheme classified partnerships in terms of area of activity, for example direct delivery of health services; health promotion; continuing professional development; under/post graduate education; support services (facilities/equipment/management); research [2]. We aimed to build on this since area of activity gives little information about partnership processes and possible outcomes. As an alternative we have grouped partnerships together by their intended impact (level of impact of LMIC partner and focus on individual/organisational development); approach to health systems strengthening (training/training and infrastructure; generic/specialist) and relationships (relative scope of influence between partners; mode of delivery – with an NGO partner or not). Among other inferences, this typology suggests one way to predict partnership sustainability based on the extent to which a partnership invests in building organisational capacity and generic skills. This is a first step in generating questions about partnership effectiveness that may be answered through evaluation. Our prediction makes a number of assumptions and Table 7 relates these to two recent evaluations of health partnership effectiveness.

**Table 7:** Links between the typology suggested in this paper and effectiveness within health partnerships

Theme	Description	Links to what is known about effective partnerships
Impact	Developing individual/organisation capacity	Movement of staff is a significant challenge to the sustainability of partnerships so organisational level input is associated with sustainability [6].
	Absolute scope of influence of the LMIC partners. (local/regional/national/ international)	Involvement of partners at many levels of the health care infrastructure is important for effective health systems strengthening and involvement of higher level organisations such as the Ministry of Health is important for sustainability [6].
Approach	Training/infrastructure	Training can be considered to be ‘gap filling’ rather than enhancing institutional capacity if the training infrastructure is not also developed [2].
	Generic/specialist	Improving one element of a health system may require multiple different interventions by building human resource capacity as well as infrastructure capacity. Focus on generic capacities may be required before a focus on specialist capacities [6].
Relationships between partners	Equality of influence	Communication between partners as equals is associated with improved sustainability [6].
	Direct delivery or via third organisation	Involvement of a third party might complicate or enhance effective communication.

We searched the published literature for alternative approaches to health partnership typologies and while we identified none for international health partnerships we identified two used to classify health partnerships within a single country. Table 8 maps our classification to this work and identifies context and external relations as important additional categories. Our work has few references to context and we feel this is an important omission in our typology. Our data set is small, so inferences from statistical testing should be interpreted with caution due to their low statistical power.

**Table 8:** Comparison of approaches to health partnership classification

	Edwards, 2015	Mitchell and Shortell, 2000 [7]	Ling, 2000 [8]
Impact	Individual/organisation capacity development	Differentiation – number of different activities, services, goals	Scale and boundaries
	Level of influence of the LMIC partners.	Partnership composition including size	Partnership members
		Centrality – importance and influence of partnership	
Approach	Training/infrastructure	The nature of the problem addressed	
	Generic/specialist		
Relationship between partners	Equality of influence	Coordination and integration – mechanisms of working together	Links between partners
	Direct delivery or via third organisation	Accountability	
Context		Alignment – nature of interactions between the partnership and external organisations	Fit with existing institutional architecture. Maturity of relationships. Resource dependency

### Limitations

The data set could have been subject to selection bias as it consisted entirely of proposals that were successful in their applications for funding. Reasons for rejection include non-eligibility; lack of information on application; inappropriate clinical or contextual relevance, unrealistic ambition and feasibility; size of grant not appropriate for level of activity proposed. The only data available to us was that in the funding applications. Some applications were more detailed than others, which could have resulted in misclassification of partnerships where intended activities were not fully described in the application. This work used data on health partnership set-ups and approaches, the next phase will be to develop a theory of change to analyse the extent to which partnerships were able to implement their plans and achieve their objectives.

## Conclusions

There has been no typology of health partnerships to date that goes beyond area of activity. We propose a typology of health partnerships with categories to describe impact, approach and relationship between partners. There is a lack of published evidence on the effectiveness of institutional partnerships as a strategy for health systems strengthening. This classification is intended to facilitate comparison between partnerships as an aid to evaluation. It is the first phase of a larger project where we will use the typology to select three different partnerships. For each of these we will develop a theory of change and model evaluation plans with the intention of comparing processes and outcomes.

## Additional files

Additional file 1:
**Impact and approach.** Table with data used for chi-squared test for an association between the variables absolute scope of influence and strategy for capacity building. (DOCX 22 kb) Additional file 2:
**Absolute and relative scope of influence.** Table with data used for chi-squared test for an association between the variables absolute scope of influence and relative scope of influence. (DOCX 22 kb)

## Abbreviations

HPS: Health partnership scheme
LMICs: Low and middle income countries
NHS: National Health Service
THET: Tropical Health Education Trust
UK: United Kingdom
