# Supplementary material for: Towards a simple typology of international health partnerships
Source: Global Health. 2015 Dec 15;11:49. doi: 10.1186/s12992-015-0132-x (PMC4681029; doi:10.1186/s12992-015-0132-x)
Supplement: Additional file 1: — Impact and approach. Table with data used for chi-squared test for an association between the variables absolute scope of influence and strategy for capacity building. (DOCX 22 kb) [file 12992_2015_132_MOESM1_ESM.docx]

**Impact and approach**

|  | International | National | Regional | Local | Total |
| --- | --- | --- | --- | --- | --- |
| Education & training | 4 (28.6%) | 10 (52.6%) | 7 (41.2%) | 2 (50.0%) | 23 |
| Education, training & infrastructure | 10 (71.4%) | 9 (47.4%) | 10 (58.8%) | 2 (50.0%) | 31 |
| Total | 14 (100%) | 19 (100%) | 17 (100%) | 4 (100%) | 54 |

71% of international partnerships provided training and infrastructure compared to 47% of national, 59% of regional and 50% of local ones. We hypothesised that international institutions would have a better sense of the importance of infrastructure and would be more likely to use a training and infrastructure approach. A chi-squared test for an association showed there was no statistical evidence of an association between absolute scope of influence and strategy for capacity building. X^2^ =2.0, P = 0.57
