# Supplementary material for: Towards a simple typology of international health partnerships
Source: Global Health. 2015 Dec 15;11:49. doi: 10.1186/s12992-015-0132-x (PMC4681029; doi:10.1186/s12992-015-0132-x)
Supplement: Additional file 2: — Absolute and relative scope of influence. Table with data used for chi-squared test for an association between the variables absolute scope of influence and relative scope of influence. (DOCX 22 kb) [file 12992_2015_132_MOESM2_ESM.docx]

**Absolute and relative scope of influence**

|  | International/National | Regional/Local | Total |
| --- | --- | --- | --- |
| Equal | 19 (65.5%) | 14 (77.8%) | 33 |
| Unequal | 10 (34.5%) | 4 (22.2%) | 14 |
| Total | 29 (100%) | 18 (100%) | 47 |

We hypothesised that relative scope of influence is more likely to be equal for partnerships where the LMIC partner has international or national absolute scope of influence compared to those with regional or local scope. A chi-squared test for an association showed there was no statistical evidence for this, X^2^ = 0.8, P=0.37
